# Molecular Identification of Species Caused Cutaneous Leishmaniasis in Southern Zone of Iran

**Published:** 2019-06-24

**Authors:** Afshin Barazesh, Mohammad Hossein Motazedian, Moradali Fouladvand, Gholamreza Hatam, Saeed Tajbakhsh, Sepideh Ebrahimi, Danial Purkamal

**Affiliations:** 1 Department of Microbiology and Parasitology, Faculty of Medicine, Bushehr University of Medical Sciences, Bushehr, Iran; 2 Department of Parasitology and Mycology, School of Medicine, Shiraz University of Medical Sciences, Shiraz, Iran; 3 The Persian Gulf Marine Biotechnology Research Center, Bushehr University of Medical Sciences, Bushehr, Iran; 4 Diagnostic Lab of Leishmaniasis, Valfajr Health Center, Shiraz University of Medical Sciences, Shiraz, Iran

**Keywords:** Cutaneous leishmaniasis, *Leishmania*, *Crithidia*, Iran

## Abstract

**Background:**

*Leishmania major* and *Leishmania tropica* are two main species causing cutaneous leishmaniasis (CL) in Iran. Recently, *Crithidia* spp. has also been reported in the wound of patients with CL. In this study, we determined the species causing CL in the southern of Iran and the role of *Crithidia* spp. in creating skin ulcers.

**Methods:**

In this cross-sectional study from Apr to Sep 2016, 66 patients with CL referred to Diagnostic Lab of Leishmaniasis, Valfajr Health Center, Shiraz, Iran, were selected. After DNA extraction from the Giemsa stained smears, all samples were amplified in two separate steps using specific primers, firstly, to differentiate *Leishmania* species and then to identify *Crithidia* spp.

**Results:**

Two species *L. major* and *L. tropica* were responsible for 60 and 6 cases, respectively. Moreover, in two patients, mixed infection with *Crithidia* was confirmed. In mix infection cases, the morphology of the cutaneous ulcers was not different from the wounds of other patients.

**Conclusion:**

*Leishmania major* is responsible for the most common CL in southern Iran. In addition, in two patients with *L. major* and *L. tropica*, mix infection with *Crithidia* was confirmed. The potential role of *Crithidia* as the main factor for CL and the probability of this parasite to have synergistic effects on *Leishmania*, as a hypothesis, requires more comprehensive researches on the ambiguity of this protozoon.

## Introduction

Cutaneous leishmaniasis (CL) is one of the six important diseases in tropical and subtropical regions which WHO has recommended and supported researches on its various aspects (1, 2).

The factor causing CL is various species of bloody-tissue protozoan parasites called *Leishmania* (3). This disease has distributed to all continents of the world except Australia and it can be seen in 88 countries. Annually, 1–1.5 million people in the world become infected with *Leishmania* and a population of about 350 million people are at risk for this disease (4, 5). About 90% of CL cases worldwide are reported from seven countries: Afghanistan, Algeria, Brazil, Iran, Peru, Saudi Arabia and Syria (6) which among these countries, Iran and Saudi Arabia have the highest incidence of the disease (7).

About 20,000 CL cases are reported annually from different parts of Iran estimated that the real rate is several times higher (8). In recent years, the prevalence CL in Iran, especially in the southern regions, has been growing gradually and alarmingly and is endemic in 17 out of 31 provinces of Iran (9), as the number of positive cases has increased from 1560 in 1991 to 3861 in 2001 (10, 11). In an epidemiological survey about CL in Iran during 2013–2015, CL is an endemic disease in Fars Province, and it annually infects numerous people (12). In general, provinces such as Fars and Isfahan with an incidence of 1.66 cases per 1,000 people, have the highest incidence of the disease in the country (13).

*Leishmania major* and *L. tropica* are two main species causing CL in Iran (9), which have a different distribution in different parts of the country (14).

In recent studies, besides these two species, some trypanosomatidal protozoa including *Herpetomonas* spp., *Leptomonas* spp. and *Crithidia* spp. have also been reported in the culture of cutaneous ulcers (15–17). *Crithidia* spp. are classified in Kinetoplastida order and Mastigophora subphylum and are often referred to as insects’ parasites (16). Two species (*Crithidia lucilia* and *Crithidia fasciculata*) are also considered to be infectious agents of the *Leishmania* culture medium (18, 19). However, the interesting thing is that the patients who the *Crithidia* spp. have been isolated from their wounds are often drug resistant to various drugs, and their cutaneous ulcers sometimes remain for 2–3yr and become chronic (17), which is probably due to the presence of *Crithidia* alone or simultaneously with *Leishmania* spp. as mix-infection in these groups. In Iran, *Crithidia* spp. has been reported frequently from patients suspected of drug-resistant cutaneous leishmaniasis and the genomic sequence of the ribosomal region has proven its belonging to the genus *Crithidia* (17). In another study, *Crithidia* was isolated from the spleen and foot-pad of *Tetera indica* in Fars Province, Iran which considers the presence and role of this protozoon in infecting vertebrate hosts (20). *Crithidia* spp. are able to present simultaneously with some trypanosomatidal parasites such as *Leishmania* spp. and be transferred to different hosts (21). For the first time reported that *C. deanei* has the ability to infect mouse fibroblast cells (22). In the following in 2010, it concluded the ability of this species to cause infections in BALB/c (23).

Since the establishment of a specific species of *Leishmania* parasite in a geographical region depends on several factors such as the presence of a specific vector and a suitable reservoir (24), the determination of the dominant species in an endemic or hyper-endemic region can be important for adopting an appropriate strategy to control vectors and reservoirs. Furthermore, identification of species is essential for more effective treatment of the patients due to the fact that the response to treatment varies in different species (13). Therefore, considering the high incidence of CL and its health importance, we decided to determine the species causing CL in the southern region of Iran and the role of *Crithidia* spp. in creating skin ulcers by using the molecular methods.

## Materials and Methods

### Sample Preparation

This study was approved by the Ethics Committee of the Bushehr University of Medical Sciences (Ethics Committee code: IR.BPUMS.REC.1395.125) and informed consent was signed by each of the patients.

In this cross-sectional study, in a period of 6 months from Apr to Sep 2016, patients with cutaneous ulcers referred to the Diagnostic Lab of Leishmaniasis, Valfajr Health Center, Shiraz, Iran were tested. Smears of them from exudates of margin of the wounds were prepared and stained with Giemsa. Finally 66 patients with CL were selected. Then a questionnaire containing demographic information and some of the variables such as age, sex, etc. were completed separately for each patient.

### DNA Extraction

DNA was extracted from the Giemsa stained smears using the commercial kit (Favorgen Biotech Corp, Cat No. FABGK001, Taiwan). Briefly, the smears on the slides were scratched and collected by lysis buffer into the microtubes and after the addition of proteinase K, incubated initially for 1 h at 60 °C and then 10min at 70 °C. By addition of absolute ethanol and transferring the samples to the column, the rest of the process was carried out according to the kit’s manufacturer’s protocol.

### PCR and Gel Electrophoresis

All extracted DNA samples were PCR-amplified in two separate steps. First, a pair of primers LIN4R and LIN17 were used to detect and differentiate of three species *L. major*, *L. infantum* and *L. tropica*, and in the next step, in order to identify *Crithidia* spp., CRF and CRR primers were used in this purpose. Further details about the sequence of primers used and the PCR programs adjusted for the amplification of both genomic pieces, has been presented in Table 1.

**Table 1.** Characteristics of primers and temperature patterns used in PCR tests

**Genus**	**Primers**	**Sequence**	**Program**			

			**Processes**	**Cycles**	**Temp**	**Time**
***Leishmania***	LIN4R (F)	5′- GGG GTT GGT GTA AAA TAG GG -3′	**P. denaturation**	1	95 °C	5min
			**Denaturation**		94 °C	30sec
	LIN17 (R)	5′- TTT GAA CGG GAT TTC TG -3′	**Annealing**	35	52 °C	30sec
			**Extension**		72 °C	45sec
			**Final Extension**	1	72 °C	8min
***Crithidia***	FCR (F)	5′- TCC ATG TGC GAG GAC AAC GTG CT -3′	**P. denaturation**	1	94 °C	3min
			**Denaturation**		95 °C	30sec
	RCR (F)	3′- CGC GTC GTT GAT GAA GTC GCT -5′	**Annealing**	30	62 °C	30sec
			**Extension**		72 °C	45sec
			**Final Extension**	1	72 °C	5min

PCR products were separated on 1.2% agarose gel and TAE (Tris, Acetate, and EDTA) buffer, and the obtained bands were detected by a UV detector (Bio-Rad, USA)

### Statistical analysis

The results and data of the questionnaires were analyzed using SPSS software version 18 and chi-square test and P-values. At levels < .05, the P*-*values were considered as statistically significant.

## Results

**Fig. 1.**
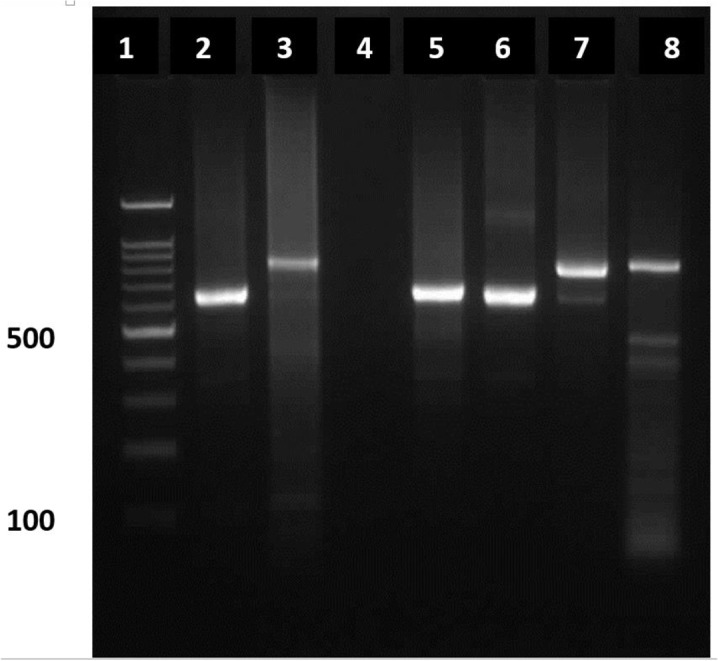
Electrophoresis of PCR products using LIN4R and LIN17 primers on agarose gel 1.2% to differentiate *Leishmania* species**.** Lane 1: Molecular weight marker**,** Lane 2: Positive control for *L. major*, Lane 3: Positive control for *L. tropica*, Lane 4: Negative control**,** Lane 5, 6: Positive samples for *L. major*, Lane 7, 8: Positive samples for *L. tropica*

**Fig. 2.**
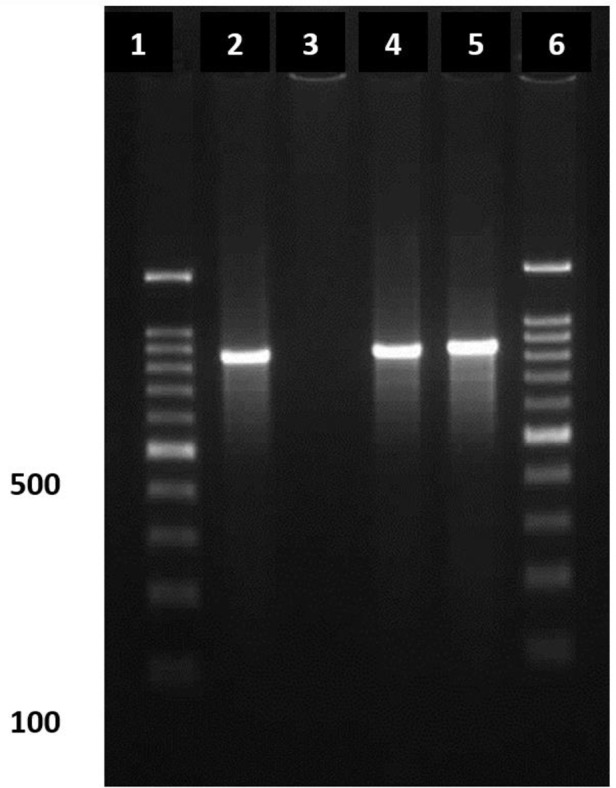
Electrophoresis of PCR products using CRF and CRR primers on agarose gel 1.2% to identify *Crithidia* spp. Lane 1: Molecular weight marker**,** Lane 2: Positive control for *Crithidia* spp**,** Lane 3: Negative control**,** Lane 4, 5: Positive samples for *Crithidia* spp., Lane 6: Molecular weight marker

Of the 66 patients, *L. major* and *L. tropica* were responsible for 60 and 6 cases, respectively (Fig. 1). Moreover, in two patients with *L. major* and *L. tropica*, mix infection with *Crithidia* was confirmed (Fig. 2).

There was no significant relationship between the disease-causing species and the factors such as the number, appearance, and location of the wounds in the body, the residence as well as the age and gender of the patients (Table 2).

**Table 2.** Demographic features of the patients with CL and the relationship of disease-causing species and some variables

**Variables**	**Species**			**P-value**

	***L. major* n (%)**	***L. tropica* n (%)**	***Crithidia*+*Leishmania* n (%)**	
**Sex**				
Male	33 (50)	2 (3)	1 (1.5)	0.78
Female	26 (39.4)	3 (4.5)	1 (1.5)	
**Age**(yr)				0.73
< 10	6 (9.1)	1 (1.5)	0 (0)	
10–20	9 (13.6)	0 (0)	0 (0)	
20–30	9 (13.6)	1 (1.5)	1 (1.5)	
30–40	11 (16.7)	2 (3)	0 (0)	
> 40	24 (36.4)	1 (1.5)	1 (1.5)	
**Residence**				0.30
City	46 (69.7)	5 (7.6)	1 (1.5)	
Village	13 (19.7)	0 (0)	1 (1.5)	
**City**				0.99
Shiraz	41 (62.1)	5 (7.6)	2 (3)	
Marvdasht	1 (1.5)	0 (0)	0 (0)	
Daryun	7 (10.6)	0 (0)	0 (0)	
Kharameh	1 (1.5)	0 (0)	0 (0)	
Tiun	2 (3)	0 (0)	0 (0)	
Meimand	2 (3)	0 (0)	0 (0)	
Maharlou	1 (1.5)	0 (0)	0 (0)	
Sharifabad	4 (6.1)	0 (0)	0 (0)	
**Number of wounds**				0.53
1	23 (34.8)	3 (4.5)	1 (1.5)	
2–5	22 (33.3)	2 (3)	0 (0)	
> 5	14 (21.2)	0 (0)	1 (1.5)	
**Duration the advent of the wounds**				0.64
1–4 wk	22 (33.3)	1 (1.5)	1 (1.5)	
4–8 wk	24 (36.4)	2 (3)	0 (0)	
> 8 wk	13 (19.7)	2 (3)	1 (1.5)	

In two patients with mix infection *(Leishmania* and *Crithidia*), the morphology of the cutaneous ulcers was not different from the wounds of the other patients.

## Discussion

Cutaneous leishmaniasis is one of the vector-borne diseases and is caused by various species of *Leishmania* spp. (3, 25). Establishment of a specific species of this parasite in any geographical region depends on several factors such as the presence of appropriate reservoir and vector, and determining the dominant species in each area can be very important in controlling the life cycle as well as the more effective treatment of the disease (13).

The isolation and mass cultivation of parasite in order to determine the species is not only very time-consuming but also is expensive (26, 27), therefore, in the present study, Giemsa stained slides were used for this purpose to reduce the costs and overcome the problem.

The success of DNA extraction from these smears has already been studied (26). Furthermore, the primers used for PCR in various studies were able to identify the genus, so to determine the parasite species, the researchers had to develop advanced molecular methods, such as PCR-RFLP or sequencing. In this study, we used the primers previously designed based on the variable part of DNA minicircles in kinatoplast (13, 28, 29). These primers have the ability to identify three species so that by electrophoresis of the PCR product, *L. major*, *L. infantum* and *L. tropica* create a specific band in the 650, 720 and 760 bases, respectively. The specificity of 100% has been reported for PCR tests using primers LIN4R and LIN17 to detect *Leishmania* in atypical cases (13, 30).

In some endemic and hyper-endemic regions of Iran *L. major* and in other areas, *L. tropica* plays a role in causing disease, although, in southern regions such as Fars Province, considered as one of the most important foci in Iran, both species are listed as endemic factors (24, 31). Recently, alongside these two dominant parasites, *Crithidia* spp. has been reported from cultivation of cutaneous ulcers of patients (15, 16).

In this study, in order to determine the role of *Crithidia* spp. in cutaneous lesions, samples were again amplified with two specific FCR and PCR primers. In two CL cases, there was a mix infection by *Leishmania* and *Crithidia* and, as shown in Fig. 2, a band in 850 bases characterizes this parasite (Fig. 2). *Crithidia* spp. are classified in flagellate subphylum and are often considered as insect parasites (16). Since the sequence of their rRNA (Ribosomal RNA) gene is very close to *Leishmania*, they are usually associated with together (16, 18). *Crithidia* spp. are considered as infectious agents of the *Leishmania* culture medium. *Crithidia lucilia* and *C. fasciculata* are two important species of this genus (18).

Over the past 20 years, various methods have been developed to determine the different subspecies of *Leishmania* and *Crithidia*, as well as to study the molecular diversity and the interaction between this parasite and the host (32–34). In recent reports, besides two *L. major* and *L. tropica* species, *Crithidia* spp. has also been proven in the wound of patients with CL, and interestingly, even in some of these reports, *Crithidia* has been reported as the only factor isolated from the wound of mentioned patients. In a study to investigate the polymorphism of two species *L. tropica* and *L. major* in the central and desert regions of Iran, *Crithidia* has been isolated from 6 of 215 and 3 of 125 patients with cutaneous ulcers in Isfahan and Bam, respectively. Molecular analysis of *Crithidia*-positive samples using BLAST software has shown that their sequence is 97 % similar to *C. fasciculata* and 90% to *C. lucilia* (15).

## Conclusion

The main cause of CL in southern Iran is primarily *L. major* and then *L. tropica*. Moreover, in two patients with *L. major* and *L. tropica*, mix infection with *Crithidia* was confirmed. And more importantly, the potential role of *Crithidia* as the main cause of the cutaneous ulcers or the probability to have synergistic effects on *Leishmania* cutaneous ulcers, as a hypothesis, needs more comprehensive researches on the ambiguity of this protozoon.
